# Juvenile Xanthogranuloma as Differential Diagnosis of a Vulvar Mass: A Case Report

**DOI:** 10.1055/s-0042-1743159

**Published:** 2022-04-19

**Authors:** Isabel Bada Bosch, Agustín del Cañizo, Minia Campos-Domínguez, Javier Ordoñez, María Dolores Blanco Verdú, María Fanjul, Laura Pérez-Egido, Juan Carlos de Agustín

**Affiliations:** 1 Department of Pediatric Surgery, Hospital General Universitario Gregorio Marañón, Madrid, Spain; 2 Department of Pediatric Dermatology, Hospital General Universitario Gregorio Marañón, Madrid, Spain

**Keywords:** vulvar mass, juvenile xanthogranuloma, rhabdomyosarcoma

## Abstract

Vulvar masses in children are an unusual finding but their differential diagnosis is extensive. In case of solid masses, rhabdomyosarcoma (RMS) must always be considered due to the fact that it is the most common tumor in external genitals during childhood. However, RMS has a radiological appearance very similar to juvenile xanthogranuloma (JXG). We present a 16-month-old girl with a 2 cm solid mass on her left labia majora, with four overlying cutaneous papules. After imaging tests, an excisional biopsy was programmed due to high malignancy suspicion. Histopathology of the mass and one of the papules was diagnostic for JXG. After a 12-month follow-up, the patient shows no signs of relapse or complication. Deep JXG is an uncommon entity in childhood and exceptional in the genital area. Therefore, it must be included in the differential diagnosis of a solid vulvar mass, especially if accompanying yellowish xanthomatous cutaneous lesions are present.

## Introduction

External genitalia masses in girls are rare. Differential diagnosis is challenging as it is very extensive and not of common knowledge. There are four main etiological groups 1 : congenital malformations or remnants of embryonic structures, hormonal disruptions, benign masses, and malignant tumors. Regarding malignant masses, rhabdomyosarcoma (RMS) must be always ruled out. We present a case of a vulvar mass in a 16-month-old girl that was resected after radiological diagnosis of a malignant tumor that, after histopathological analysis, was diagnosed as juvenile xanthogranuloma (JXG).

## Case Report

**Fig. 1:**
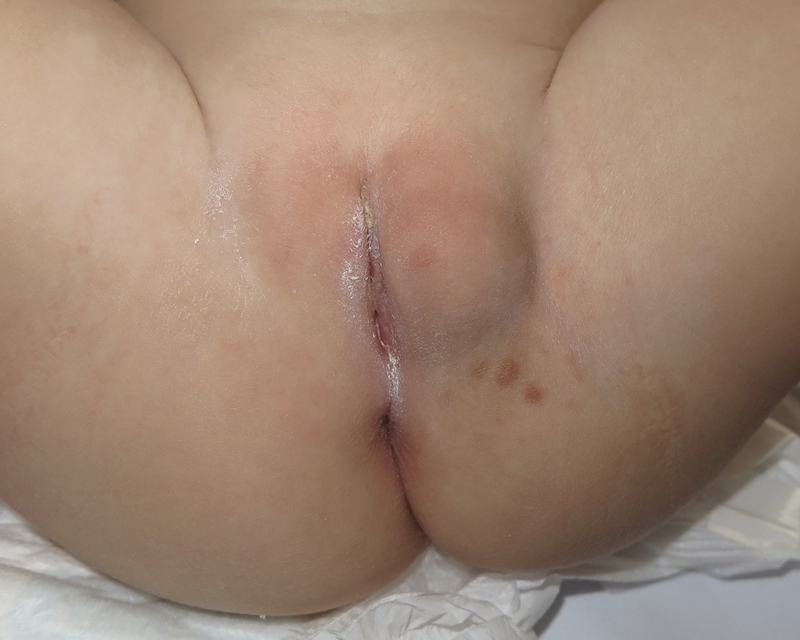
Vulvar mass on the left labium majus surrounded by four cutaneous papules.

A 16-month-old girl with a 2.5-month history of an enlarging vulvar mass on her labia majora was referred to our center by her pediatrician. Family members reported significant growth in the last 24 hours. Otherwise, the patient did not show fever or other symptoms. The mass was neither painful, fluctuant nor discharged purulent material at compression. Examination revealed a hard, well-defined, nonerythematous, nonfluctuating tumor on her left labium majus as well as bilateral inguinal lymphadenopathies. Four erythematous papules were seen on the overlying skin ( Fig. 1 ).

**Fig. 2:**
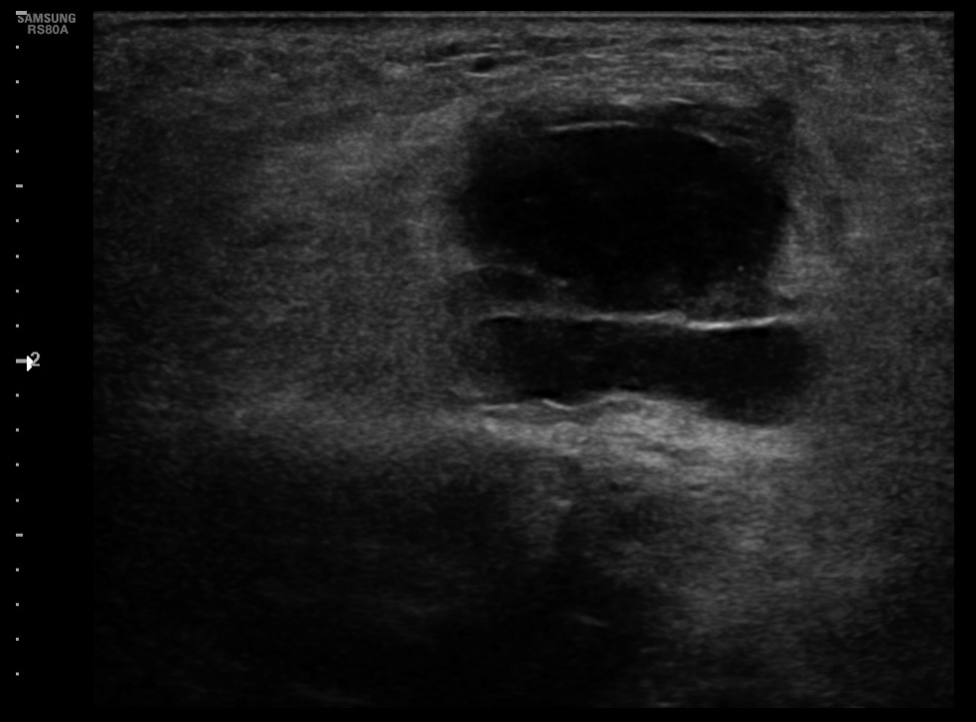
Ultrasound scan of hypoechoic bilobulated mass with heterogeneous content and perilesional edema.

A perineal ultrasound scan described a poorly defined 28 × 17 × 24 mm cystic image with heterogeneous content associated with perilesional edema ( Fig. 2 ).

**Fig. 3:**
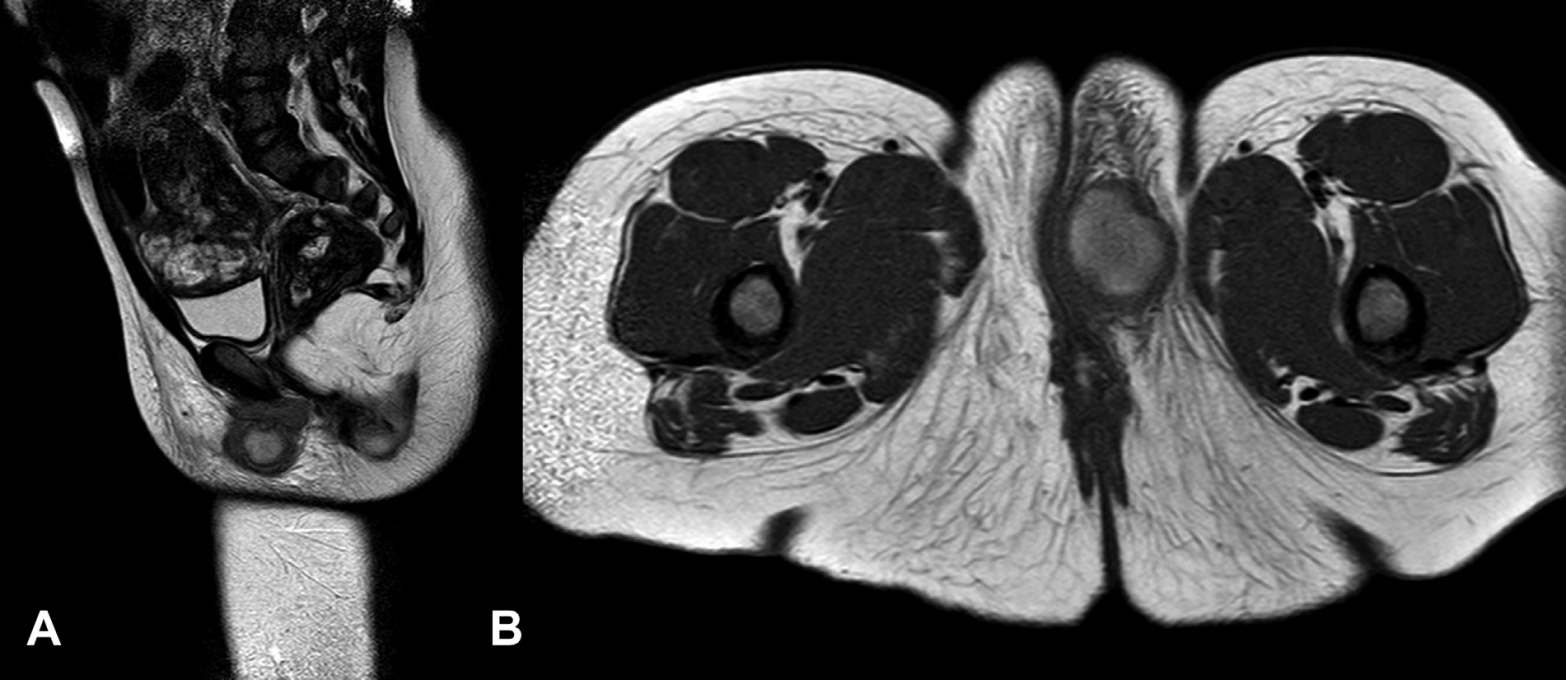
Sagittal ( **A** ) and axial ( **B** ) sections, T2-weighted magnetic resonance imaging showing a hyperintense rounded mass, encapsulated with a necrotic central component and ring enhancement.

Contrast-enhanced magnetic resonance imaging (MRI) was performed ( Fig. 3 ). It showed a 26 × 18 × 20 mm bilobulated lesion with two components: an ovoid one resting on the lateral wall of the vagina and another deeper solid tubular component. The first component had a necrotic center and its walls showed enhancement after contrast administration. The second component was in contact with the muscular plane of the puborectalis, transversus perinei, bulbospongiosus, and ischiocavernosus. The lesion showed infiltrative margins with subcutaneous cellular tissue striation but without bone involvement. Besides, bilateral inguinal lymphadenopathies showing signs of metastatic involvement were described. The mass was defined as a possible RMS.

Given the suspected malignancy, an open biopsy was scheduled. In the lithotomy position, a left parasagittal incision was made on the left labium majus, revealing a 2.5 cm encapsulated tumor attached to bone, with a cranial extension toward the puborectalis muscle. Due to the good delimitation of the lesion and the absence of macroscopic infiltration, an excisional biopsy was performed. The cutaneous satellite lesions were biopsied at the same procedure. The patient was discharged on postoperative day 7 with a mild surgical wound hematoma in resolution.

**Fig. 4:**
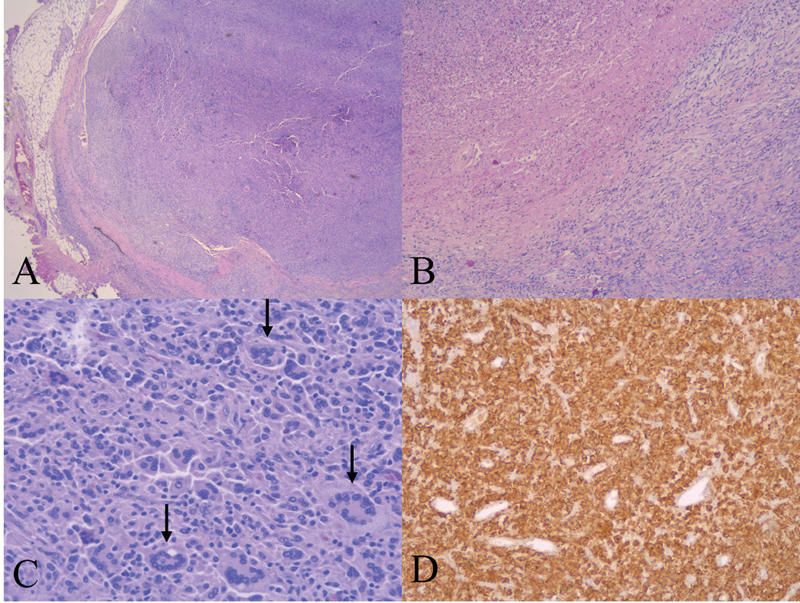
Histological images of ( **A** ) encapsulated, noninfiltrative tumor, surrounded by normal adipose tissue, ( **B** ) histiocytic cells (lower part of the image) and necrosis areas (middle area), ( **C** ). histiocytes intermixed with multinucleated Touton cells (arrows), ( **D** ) immunohistochemistry for CD68.

Histopathological examination ( Fig. 4 ) showed a nodule with a central necrosis area that represented 40% of the mass volume. It was surrounded by a chronic inflammatory infiltrate with histiocytic and lymphocytic inflammatory cells. There were two populations of histiocytes, cells with large poorly demarcated eosinophilic cytoplasms with isomorphic nuclei and prominent nucleoli mixed with foamy histiocytes. Frequent multinucleated Touton giant cells were identified. There were no signs of cellular or nuclear atypia, with few mitoses. Immunohistochemistry examination was positive for CD163, CD68, and factor XIIIa; negative for S100 and CD1a. The surgical specimen was defined as JXG. The skin lesions were also histiocytic, with the same pattern of immunohistochemical expression.

After having ruled malignancy out, the patient did not receive any adjuvant treatment. She was referred to the ophthalmology clinic to rule out ocular involvement, which was absent. An abdominal ultrasound was performed without abnormal findings. She is currently followed at the outpatient pediatric oncology and pediatric surgery clinics, without any complications or signs of recurrence after a 12-month follow-up.

## Discussion

Vulvar tumors, although infrequent, include a very wide, and in many cases unknown, differential diagnosis.

Regarding benign masses, they are mostly mesenchymatous: lipoma, 2 lipoblastoma, 3 4 fibroma, 5 6 hamartoma, 7 8 neurofibromas, 1 rhabdomyoma, hemangioma, or lymphatic malformations. Other less frequent benign tumors that have been described are plexiform schwannoma, 9 fibrous angiomatoid histiocytoma, 10 or granular cell tumor. 11

Regarding malignant tumors, the most common lower genital neoplasm in children is RMS. 12 Another malignant tumor, although exceptional in this location, is endodermal sinus tumor. Scherr et al 13 described a case of a primitive neuroectodermal tumor in the vulvar region in a 10-year-old patient. There are also reported cases of adulthood tumors diagnosed in girls such as angiomyxoma, angiomyofibroblastoma, cellular angiofibroma, or stromal fibroepithelial polyp. 5

JXG is not usually included the differential diagnosis of vulvar masses. This entity, although unusual, is the most frequent non-Langerhans histiocytosis. It is characterized by single or multiple papules or nodules, initially pink but later turning yellowish. Although most of them are cutaneous, there are subcutaneous and visceral variants. They are typically found in infants and young children, most of them appearing during the first year of life. Its most frequent locations are head, neck, and trunk. Extracutaneous involvement is rare (4% of cases), eye being the most commonly affected organ. Their natural tendency is toward long-term regression; therefore, they usually do not require surgery and it is reserved for atypical cases.

Being the upper body its most common location, genital lesions do not usually raise suspicion of this entity. There are very few published articles reporting this location. After an exhaustive bibliographic research, we found a total of 12 cases in children: three patients with lesions on the penis, 14 15 16 two testicular, 17 18 and one scrotal. 19 As for women, five cases of JXG in the labia majora 20 21 22 23 and one case in the clitoral hood of a 5-week-old girl 24 have been described. These five patients were managed exactly the same as in our case; diagnostic doubts forced surgical excision that was diagnostic as well as curative.

Our patient presented with a short-time history of a vulvar mass without the typical macroscopic characteristics of a JXG, so this diagnosis was not considered at first. The initial diagnostic test for a vulvar mass is a soft tissue ultrasound. Given the impossibility of ruling out a RMS, we performed an MRI to characterize the mass, which, in this case, showed malignancy data. RMS MRI image is similar to that of the JXG, both lesions are hypointense on T1-weighted images and hyperintense on T2, being invasion of other tissues the key sign to distinguish them. 14 Given the diagnostic doubt and the malignancy suspicion, obtaining a tissue sample was compulsory. Despite a similar age at presentation and radiological features, histologically both entities show a very different image. RMS shows a proliferation of monomorphic small round cells (rhabdomyoblasts), atypia, mitosis, and necrosis 23 accompanied by a zone of hypercellularity in the submucosa. 5 JXG is characterized (in its mature phase) by a diffuse tissue invasion of a histiocytic population with large eosinophilic cytoplasms, Touton giant cells, and foamy cells accompanied by a chronic inflammatory infiltrate. 25 There should not be abundant mitosis or any type of atypia within this infiltrate. After a certain period of time, evolved JXG may show signs of fibrosis. They also show a different immunohistochemical profile, the first being positive for muscle tissue markers such as desmin, myogenin, or MyoD1; 5 and JXG positive for CD68, K _i_ -M1P, anti-factor XIIIa, vimentin, and anti-CD4 but negative for CD-1a and S-100. 16 Table 1 depicts the differential diagnosis between these two entities.

**Table 1:** Differential diagnosis between JXG and RMS

	JXG	RMS
Age	Mostly <1 year	Two peaks: 2–6 years and 10–18 years
Location	Head, neck, and trunk Genitals are exceptional	Head and neck (35%) Genitourinary (25%)
Radiological image in MRI	Hypo-T1, hyper-T2 No signs of invasion	Hypo-T1, hyper-T2 Invasion, metastases, and lymphadenopathies
Histology	Histiocytes and Touton giant cells Chronic inflammatory infiltrate. Possible necrosis No mitosis nor atypia.	Monomorphic small round cells, atypia, mitosis, and necrosis Hypercellularity in submucosa
Immunohistochemistry	CD68 + , K _i_ -M1P + , anti-factor XIIIa +, vimentin +, anti-CD4+ CD-1a-, S-100-	Desmin +, myogenin +, MyoD1+
Treatment	None	Surgery, chemotherapy, radiotherapy according to clinical guidelines
Prognosis	Self-resolving. Excision is curative	Genital cases usually have good prognosis

Abbreviations: JXG, juvenile xanthogranuloma; MRI, magnetic resonance imaging; RMS, rhabdomyosarcoma.

After appropriate histological diagnosis, extracutaneous involvement must be ruled out. 26 Patients should be referred to the ophthalmologist for an eye examination. Assessment of visceral involvement is usually not required and should be reserved for multiple, infiltrating or extensive JXG cases. In those cases, abdominal ultrasound, chest X-ray, and, occasionally, bone marrow biopsy are indicated. Our patient presented a slightly infiltrating mass, but with an atypical location and presentation, so ophthalmological review and abdominal ultrasound were performed, both without pathological findings.

JXG is a benign pathology that has a tendency toward disappearance over a period of 3 to 6 years. However, its heterogeneity in morphological aspect and evolution, as well as its low prevalence, makes its diagnosis challenging. In many occasions, surgical resection ends up being required to procure histological diagnosis, as it was in our patient's case. Surgical treatment in JXG, although not essential, implies definitive resolution of the disease.

## Conclusion

Vulvar masses in childhood are infrequent and include a very wide differential diagnosis, from congenital lesions, hormonal disruptions to benign or malignant tumors.

Deep JXG is an infrequent entity. Its genital location is exceptional, with only 12 cases described in the scientific literature.

The radiological image of the RMS is similar to that of the JXG, so in cases of diagnostic doubt, a biopsy of the mass is essential, since the histological diagnosis is definitive. Although JXG is a self-resolving pathology, diagnostic doubts may force surgical excision, which is also curative.

## Notes

New Insights & the Importance for the Pediatric Surgeon In this article, we report a case of a deep vulvar juvenile xanthogranuloma in an infant. This is significant because juvenile xanthogranuloma is exceptional in this region but should be considered in the differential diagnosis of vulvar mases, which is very extensive.
